# Ultra-Low Background DNA Cloning System

**DOI:** 10.1371/journal.pone.0056530

**Published:** 2013-02-08

**Authors:** Kenta Goto, Yukio Nagano

**Affiliations:** 1 Analytical Research Center for Experimental Sciences, Saga University, Honjo, Saga, Japan; 2 Department of Applied Biochemistry and Food Science, Saga University, Honjo, Saga, Japan; Florida International University, United States of America

## Abstract

Yeast-based *in vivo* cloning is useful for cloning DNA fragments into plasmid vectors and is based on the ability of yeast to recombine the DNA fragments by homologous recombination. Although this method is efficient, it produces some by-products. We have developed an “ultra-low background DNA cloning system” on the basis of yeast-based *in vivo* cloning, by almost completely eliminating the generation of by-products and applying the method to commonly used *Escherichia coli* vectors, particularly those lacking yeast replication origins and carrying an ampicillin resistance gene (Amp^r^). First, we constructed a conversion cassette containing the DNA sequences in the following order: an Amp^r^ 5′ UTR (untranslated region) and coding region, an autonomous replication sequence and a centromere sequence from yeast, a *TRP1* yeast selectable marker, and an Amp^r^ 3′ UTR. This cassette allowed conversion of the Amp^r^-containing vector into the yeast/*E. coli* shuttle vector through use of the Amp^r^ sequence by homologous recombination. Furthermore, simultaneous transformation of the desired DNA fragment into yeast allowed cloning of this DNA fragment into the same vector. We rescued the plasmid vectors from all yeast transformants, and by-products containing the *E. coli* replication origin disappeared. Next, the rescued vectors were transformed into *E. coli* and the by-products containing the yeast replication origin disappeared. Thus, our method used yeast- and *E. coli*-specific “origins of replication” to eliminate the generation of by-products. Finally, we successfully cloned the DNA fragment into the vector with almost 100% efficiency.

## Introduction

DNA cloning is a fundamental technique for modern molecular biology. DNA cloning methods are often inefficient; thus, it is important to develop a more effective method. Yeast-based *in vivo* cloning is based on homologous recombination and is an efficient cloning method [1]–[3]. This method requires a DNA fragment of interest to be PCR-amplified with more than 20 bp of homology to the crossover region of the plasmid vector, and the vector which is digested with a restriction enzyme within the crossover region [1], [4]. Cotransformation of these DNA fragments into yeast results in cloning of the DNA fragment into the vector. This method is used in various applications: multifragment DNA cloning [2], [3], [5], [6], cloning of large DNA fragments [7], cloning of targeted regions from eukaryotic chromosomes (transformation-associated recombination (TAR) cloning) [8], and one of the procedures used to synthesize a bacterial cell [9]. In this method, the required restriction ends can be located anywhere within the crossover regions of the vector; that is, the sites at which homologous recombination occurs are not always located at the restriction ends in the plasmid vector, thus making the cloning method more flexible. Although multifragment DNA cloning and cloning of large DNA fragments are well-known advantages of yeast-based *in vivo* cloning, this flexibility can be extremely useful for routine laboratory experiments

As described previously [2], we have extended yeast-based *in vivo* cloning to experiments with frequently used plasmid vectors. These vectors, such as animal, insect, and bacterial plasmid vectors, have a backbone sequence containing an ampicillin resistance gene (Amp^r^) and a pUC origin (pUC ori), and carry no yeast replication origin. In order to convert this type of vector into the yeast/*Escherichia coli* shuttle vector, we have constructed the conversion cassette SU0, which is a linearized DNA fragment created by restriction digestion of the circular plasmid pSU0 (although we did not use this name in our previous report [2], we have used the name SU0 here to more clearly indicate that we are referring to the conversion cassette). The circular pSU0 contains the following DNA sequences in order: an Amp^r^ gene, a 2µ yeast replication origin (2µ origin), a *URA3* yeast selectable marker (*URA3*), a pUC ori, and the restriction sites for linearization. Using this cassette, homologous recombination in yeast converts the plasmid vector carrying Amp^r^ and pUC ori into a yeast/*E. coli* shuttle vector. Furthermore, cotransformation of the conversion cassette SU0 into yeast with the desired DNA fragments and this type of plasmid vector results in conversion of the vector into a yeast/*E. coli* shuttle vector and simultaneously allows cloning of the DNA fragment into the same vector. The constructed plasmids were rescued from yeast and transformed into *E. coli* to obtain sufficient plasmid for use in subsequent experiments. Although this method is highly efficient, we observed some by-products [2].

To develop a more efficient cloning method, we improved our previous methods [2] by constructing a new conversion cassette SU32. In this method, we used differential selections of plasmids based on their replication origins to eliminate the generation of by-products almost completely. The cassette contains the following DNA sequences (in order): an Amp^r^ 5′ UTR (untranslated region) and coding region, an autonomous replication sequence and centromere sequence (ARS/CEN), a *TRP1* yeast selectable marker (*TRP1*), and an Amp^r^ 3′ UTR. Thus, conversion cassette SU32 carries no *E. coli* replication origin, such as pUC ori, in contrast to our previous method [2]. Many types of vectors contain an Amp^r^ sequence. In yeast-based *in vivo* cloning, the new cassette allows conversion of this type of vector into the yeast/*E. coli* shuttle vector through the use of the Amp^r^ sequence and simultaneously allows cloning of the desired DNA fragment into the same vector. This step eliminated the generation of by-products containing only the *E. coli* replication origin. Similar to our previous method, the constructed plasmids were rescued from yeast and transformed into *E. coli* for further analysis. This step eliminated the generation of by-products containing only the yeast replication origin (e.g., circularized conversion cassette). With almost 100% efficiency, these procedures enabled us to clone the desired DNA fragment into an Amp^r^-containing vector. Thus, we succeeded in developing an “ultra-low background DNA cloning system.”

## Materials and Methods

### Strains

*E. coli* strain HST 08 Premium Electro-Cells (TAKARA BIO, Ohtsu, Shiga, Japan) were used for all electroporation experiments. Yeast strain YPH499 (*MATa ura3-52 lys2-801^amber^ ade2-101^orchr^ trp1-Δ63 his3-Δ200 leu2-Δ1*) was used for yeast-based *in vivo* cloning.

### Culture and media

Yeast growth medium was comprised of double-strength yeast extract-peptone-adenine-dextrose (2× YPAD). This medium contained 2% yeast extract, 4% peptone, 4% glucose, and 0.2 mg/mL adenine. Yeast selection was performed on synthetic complete plates lacking tryptophan. In the experiment in which pUC19 was converted into the yeast/*E. coli* shuttle vector by using the conversion cassette SU32, Luria Bertani (LB) agar containing 100 µg/mL ampicillin, 1 mM isopropyl β-D-1-thiogalactopyranoside (IPTG), and 40 µg/mL 5-bromo-4-chloro-indolyl-β-D-galactopyranoside (X-gal) was used for *E*. *coli* selection. In the experiment in which *GFPuv* or *yEGFP* was cloned into pUC19, which was simultaneously converted into the yeast/*E. coli* shuttle vector, LB agar containing 100 µg/mL ampicillin and 1 mM IPTG was used for *E. coli* selection.

### Construction of the plasmids pSU30 and pSU32

First, we constructed plasmid pSU30 (DDBJ accession number AB759085), which contains the following DNA sequences (in this order): an ampicillin resistance gene (Amp^r^) 5′ UTR and coding region (992 bp), a gentamycin resistance (Gm^r^) 3' UTR(33 bp), an autonomous replication sequence and centromere sequence (ARS/CEN; 517 bp), a *TRP1* yeast selectable marker (*TRP1*; 1,002 bp), an Amp^r^ 3' UTR (90 bp), an *Eco*RI site (6 bp), a pUC origin for *E. coli* replication (1,078 bp), and a *Bam*HI site (6 bp). To construct this plasmid, 5 DNA fragments were created, that is, an Amp^r^ 5′ UTR and coding region and a gentamycin resistance (Gm^r^) 3′ UTR, a yeast autonomous replication sequence and centromere sequence (ARS/CEN), a *TRP1* yeast selectable marker, an Amp^r^ 3′ UTR, and a pUC origin. The plasmid pDEST22 served as the template for amplification of these DNA fragments. PCR primer pairs were pSU30-1 and pSU30-2, pSU30-3 and pSU30-4, pSU30-5 and pSU30-6, pSU30-7 and pSU30-8, and pSU30-9 and pSU30-10 (40 cycles of 98°C for 10 s, 55°C for 15 s, and 68°C for 75 s using Prime STAR GXL DNA polymerase; TAKARA BIO, Ohtsu, Shiga, Japan). These primers (Table 1) were designed to provide an end homologous to that of the adjacent DNA fragment. In addition, the primer pSU30-8 was designed to provide an *Eco*RI site and primer pSU30-1 was digested to provide a *Bam*HI site. Cotransformation of these 5 DNA fragments into yeast resulted in homologous recombination at the overlapping sequences and subsequent formation of pSU30, the content of which was confirmed by DNA sequencing.

**Table 1:** Primers.

Name	Sequence
pSU30-1	5′-AACGAAAACTCACGTTAAGGGATTTTGGTCATGAGGGATCCCAGGTGGCACTTTTCGGG-3′
pSU30-2	5′-TCGGCCGGGAAGCCGATCTCGGCTTGAACGAATTGTTACCAATGCTTAATCAG-3′
pSU30-3	5′-CAATTCGTTCAAGCCGAGATCGGCTTCCCGGCCGACGGATCGCTTGCCTGT-3′
pSU30-4	5′-GGTCCTTTTCATCACGTG-3′
pSU30-5	5′-TATAATTATTTTTATAGCACGTGATGAAAAGGACCACGACATTACTATATATA-3′
pSU30-6	5′-GGCAAGTGCACAAACAAT-3′
pSU30-7	5′-GTAGTATTTATTTAAGTATTGTTTGTGCACTTGCCCTGTCAGACCAAGTTTAC-3′
pSU30-8	5′-ATTTCACACAGGAAACAGCTATGACCATGATTACGGAATTCATCAAAAAGGATCTTCAC-3′
pSU30-9	5′-CGTAATCATGGTCATAGC-3′
pSU30-10	5′-CTCATGACCAAAATCCCT-3′
pSU30-14	5′-CAGGTGGCACTTTTCGGGGAAATGTG-3′
pSU30-23	5′-ATCAAAAAGGATCTTCACCTAGATCCTTTTAAATTAAAAATGAAGTTTTAAATCAATCTAAAGTATATATGAGTAAACT-3′
pSU32-F	5′-AAAGGATCTAGGTGAAGATCCTTTTTGATGAATTCGCGCGGGATCCCAGGTGGCACTTTTCGGGGAAATGTGCGC-3′
pUCGFPF	5′-GAGCGGATAACAATTTCACACAGGAAACAGCTATGGCTAGCAAAGGAGAAGAACT-3′
PUCGFPR	5′-TTTTCCCAGTCACGACGTTGTAAAACGACGGCCAGTTATTTGTAGAGCTCATCCA-3′
yEGFPtopUC-F	5′-GAGCGGATAACAATTTCACACAGGAAACAGCTATGTCTAAAGGTGAAGAATTATT-3′
yEGFPtopUC-R	5′-TTTTCCCAGTCACGACGTTGTAAAACGACGGCCAGTTATTTGTACAATTCATCCA-3′
pGFPuv1	5′-CCGGATCATATGAAACGGCATGACTTTTTCAAGAG-3′
pGFPuv2	5′-CTCTTGAAAAAGTCATGCCGTTTCATATGATCCGG-3′

Next, we constructed plasmid pSU32 (DDBJ accession number AB759086) by removing the pUC origin from pSU30. pSU30 was digested by restriction enzymes *Eco*RI and *Bam*HI. Using this digested DNA as a template, a DNA fragment that does not carry the pUC origin was amplified by primers pSU30-14 and pSU30-23. Then, this DNA fragment was transformed into yeast with primer pSU32-F (Table 1), which links the ends of this amplified fragment. This primer was also designed to provide *Eco*RI and *Bam*HI sites, which were separated by the 5-bp linker sequence 5′-GCGCG-3′. PCR analysis confirmed the absence of the pUC origin. The plasmid pSU32 was purified from yeast for subsequent analysis.

### Preparation of conversion cassette SU32

The conversion cassette SU32 was prepared by PCR using *Eco*RI/*Bam*HI-digested pSU32 as template. For PCR amplification, the primer pair (Table 1) was pSU30-14 and pSU30-23 (40 cycles of 98°C for 10 s, 55°C for 15 s, and 68°C for 75 s using Prime STAR GXL DNA Polymerase). The amplified conversion cassette was purified by MonoFas DNA Purification Kit I (GL Sciences, Tokyo, Japan).

### PCR amplification of *GFPuv* and *yEGFP*

Open reading frames (ORFs) of green fluorescent protein variant gene (*GFPuv*) and the yeast-enhanced green fluorescent protein gene (*yEGFP*) were obtained by PCR amplification from pBAD-GFPuv (Clontech Laboratories, Mountain View, CA, USA; accession no. U62637) and pDDGFP2 (a gift from Dr. Drew). For these amplifications, custom PCR primers (pUCGFPF, pUCGFPR, yEGFPtopUC-F, and yEGFPtopUC-R) purified by polyacrylamide gel electrophoresis were purchased from Integrated DNA Technologies (Coralville, IA, USA). For the *GFPuv* cloning experiment, the *GFPuv* gene was PCR-amplified with primers (Table 1) pUCGFPF and pUCGFPR (30 cycles of 98°C for 10 s, 55°C for 5 s, and 72°C for 15 s using Prime STAR MAX DNA Polymerase; TAKARA BIO, Ohtsu, Shiga, Japan). These primers share a 35-bp overlap at their ends with the pUC19 vector. For the *yEGFP* cloning experiment, the *yEGFP* gene was PCR-amplified with primers (Table 1) yEGFPtopUC-F and yEGFPtopUC-R (30 cycles of 98°C for 10 s, 55°C for 15 s, and 68°C for 3 min using Prime STAR GXL DNA Polymerase). These primers share a 35-bp overlap at their ends with the pUC19 vector. These PCR-amplified DNA fragments were purified by MonoFas DNA Purification Kit I.

For the multifragment DNA cloning experiment, we used 2 fragments of the split *GFPuv* gene, one fragment corresponding to the nucleotides from 1 (start codon) to 260 and another fragment corresponding to the nucleotides from 226 to 720 (stop codon). Both fragments had 35-bp overlaps. The first DNA fragment was PCR-amplified with the primers pUCGFPF and pGFPuv2, and the second DNA fragment was PCR-amplified with the primers pUCGFPR and pGFPuv1 (40 cycles at 98°C for 10 s, 55°C for 5 s, and 72°C for 5 s with Prime STAR MAX DNA Polymerase). Custom PCR primers (pGFPuv1 and pGFPuv2) purified by polyacrylamide gel electrophoresis were purchased from Hokkaido System Science (Sapporo, Hokkaido, Japan).

### Yeast and *E. coli* transformation

For cloning experiments, the pUC19 vector was digested with *Eco*RI-HF (New England Biolabs, Ipswich, MI, USA) and *Xba*I (Fermentas, Vilnius, Lithuania) in the multiple cloning site (MCS). The digested vector was purified by MonoFas DNA Purification Kit I. After overnight incubation of yeast cells in 3 mL 2× YPAD broth at 30°C, they were inoculated into 20 mL 2× YPAD broth and grown at 30°C for 3 hours. The yeast cells were harvested and transformed according to Gietz and Woods [10]. However, the transformation procedure was performed at half the scale of the published method. Cells were cotransformed with the conversion cassette SU32, digested pUC19, and the PCR fragment(s) to be cloned. The molar ratio of the 3 DNA fragments was 1∶1∶1. The transformants were selected using synthetic complete plates lacking tryptophan and incubated at 30°C. We obtained about 1,000 transformants per 50 ng of digested pUC19 in both cloning experiments. For the multifragment DNA cloning experiment, the molar ratio of the 4 DNA fragments was 1∶1∶1∶1. All colonies were scraped from the plates with plating beads, and the collected yeast cells were disrupted by acid-washed glass beads (350–500 µm). Plasmids were purified from the disrupted cells using a Mini-M Plasmid DNA Extraction System (Viogene, Taipei, Taiwan). In certain instances, the rescued plasmids were digested with *Sph*I-HF (New England Biolabs). The rescued plasmids were transformed into *E. coli* HST 08 Premium Electro-Cells by electroporation and plated on agar containing 100 µg/mL ampicillin and 1 mM IPTG. The green fluorescent colonies were counted.

## Results and Discussion

**Figure 1:**
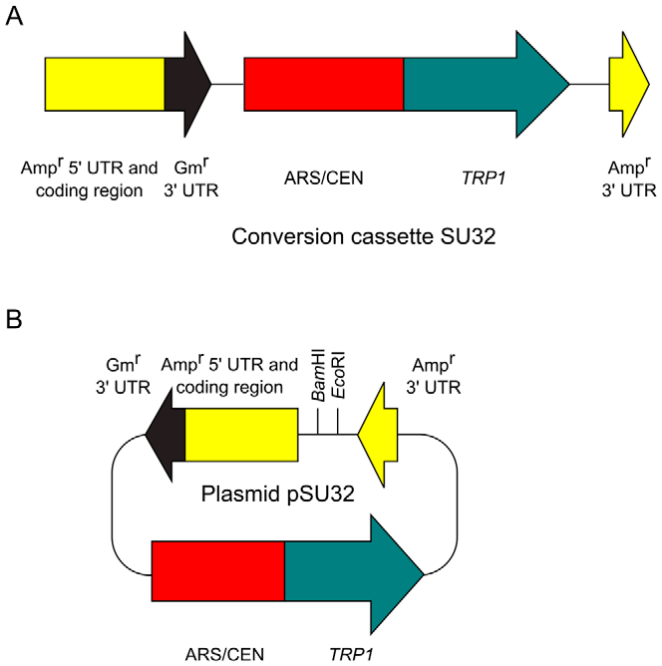
Schematic map of the conversion cassette SU32 and the plasmid pSU32. (A) Map of the conversion cassette SU32. Gm^r^: gentamycin resistance. (B) Map of the plasmid pSU32.

To develop a more efficient cloning method by improving our previous method [2], we constructed a new conversion cassette SU32 (Fig. 1A). First, we created a circular plasmid pSU32 by assembly of multiple DNA fragments in yeast (Fig. 1B). The circular plasmid pSU32 contains DNA sequences (in the following order): an Amp^r^ 5′ UTR and coding region, a gentamicin resistance gene (Gm^r^) 3′ UTR, an autonomous replication sequence and a centromere sequence (ARS/CEN), a *TRP1* yeast selectable marker (*TRP1*), an Amp^r^ 3′ UTR, an *Eco*RI site, and a *Bam*HI site. The plasmid pSU32 was extracted from yeast and digested with *Eco*RI and *Bam*HI. Conversion cassette SU32 was created by PCR, using linearized pSU32 as a template. A characteristic feature of cassette SU32 is the absence of the *E. coli* replication origin. As described below, this feature is important to eliminate the generation of by-products. Although we used the 2µ origin as a yeast replication origin in the conversion cassette SU0 [2], we used the ARS/CEN origin in the SU32 cassette because the ARS/CEN origin may allow more stable maintenance of the plasmid in yeast than the 2µ origin. We used the Gm^r^ 3′ UTR to terminate the transcription of the ampicillin-resistance gene.

**Figure 2:**
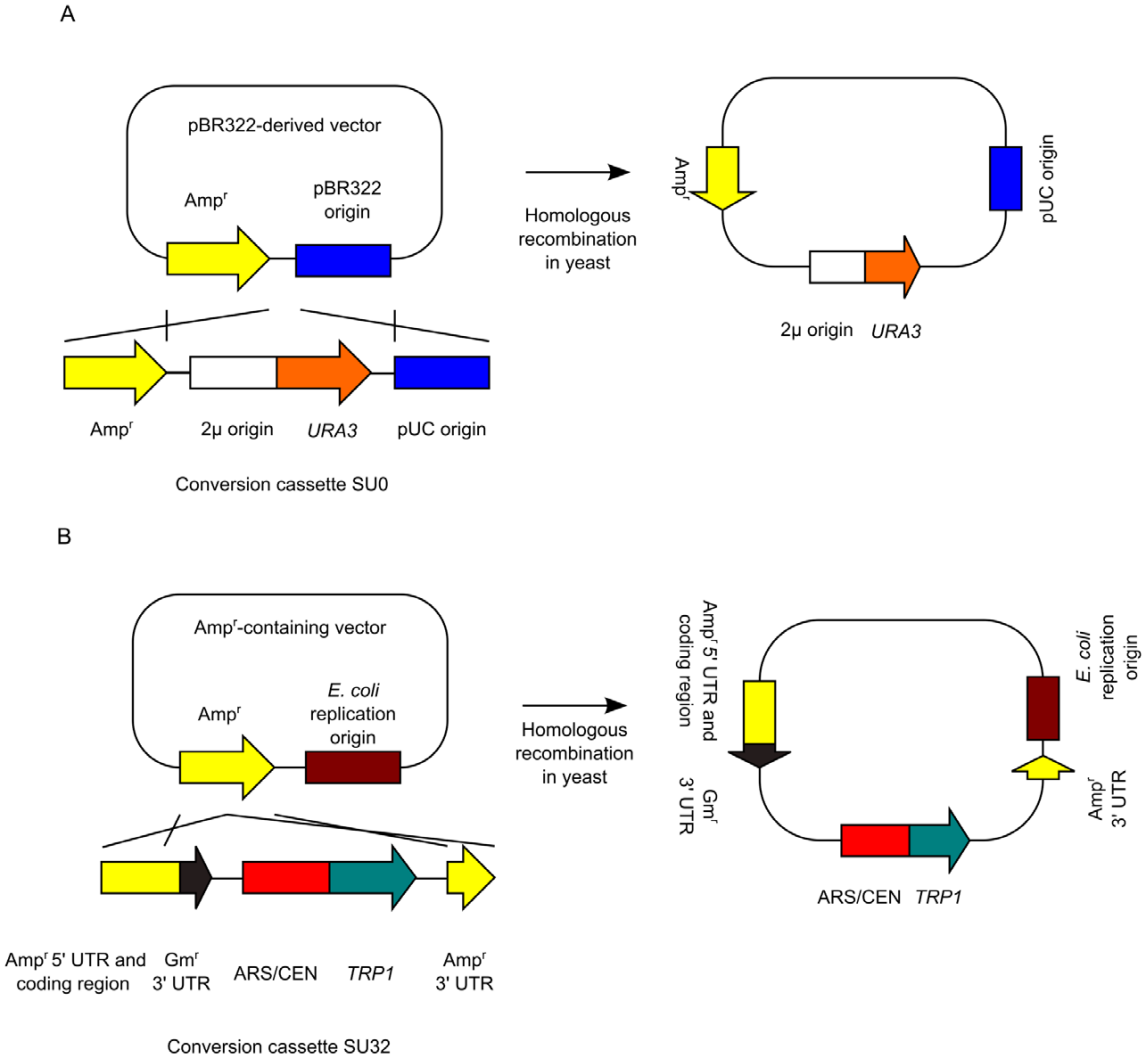
Schematic representation of the conversion of an *E. coli* plasmid vector into a yeast/*E. coli* shuttle vector with conversion cassettes. (A) Conversion of a pBR322-derived plasmid (e.g., pUC plasmid) vector into the yeast/*E. coli* shuttle vector with conversion cassette SU0 [2]. The cross represents homologous recombination. (B) Conversion of an Amp^r^-containing plasmid vector into the yeast/*E. coli* shuttle vector with the conversion cassette SU32.

Many types of plasmid vectors contain the Amp^r^ sequence. As described previously [2], we developed the conversion cassette SU0 (Fig. 2A). Conversion cassette SU0 can convert an Amp^r^-containing vector into the yeast/*E. coli* shuttle vector through the use of 2 sequences; Amp^r^ and pUC ori. In contrast, the conversion cassette SU32 can convert the Amp^r^-containing vector into the yeast/*E. coli* shuttle vector through use of only the Amp^r^ sequence (Fig. 2B) because it carries the split Amp^r^ sequence. Thus, the conversion cassette SU32 can be applied to more commonly used vectors. When we cloned the desired DNA fragment into the Amp^r^-containing vector, the new conversion cassette SU32 eliminates the emergence of by-products because we use differential selection based on the plasmid replication origins. Cotransformation of the cassette into yeast with an Amp^r^-containing vector results in conversion of the vector into the yeast/*E. coli* shuttle vector. This step eliminates the generation of by-products containing only the *E. coli* replication origin. Next, the plasmids are rescued from yeast and transformed into *E. coli*. This step eliminates the occurrence of by-products containing only the yeast replication origin. Thus, we speculated that we could eliminate the generation of by-products almost completely by using the new conversion cassette SU32.

First, to test the conversion ability of cassette SU32, we converted pUC19 into the yeast/*E. coli* shuttle vector (Fig. 2B). Yeast was transformed with cassette SU32 and circular pUC19 in a 1∶1 molar ratio. We rescued the plasmid vectors from all yeast transformants; then, the plasmid vectors were transformed into *E. coli*. Because all colonies were *lacZ*-positive, we converted pUC19 into the yeast/*E. coli* shuttle vector by using conversion cassette SU32 with 100% efficiency (data not shown).

**Figure 3:**
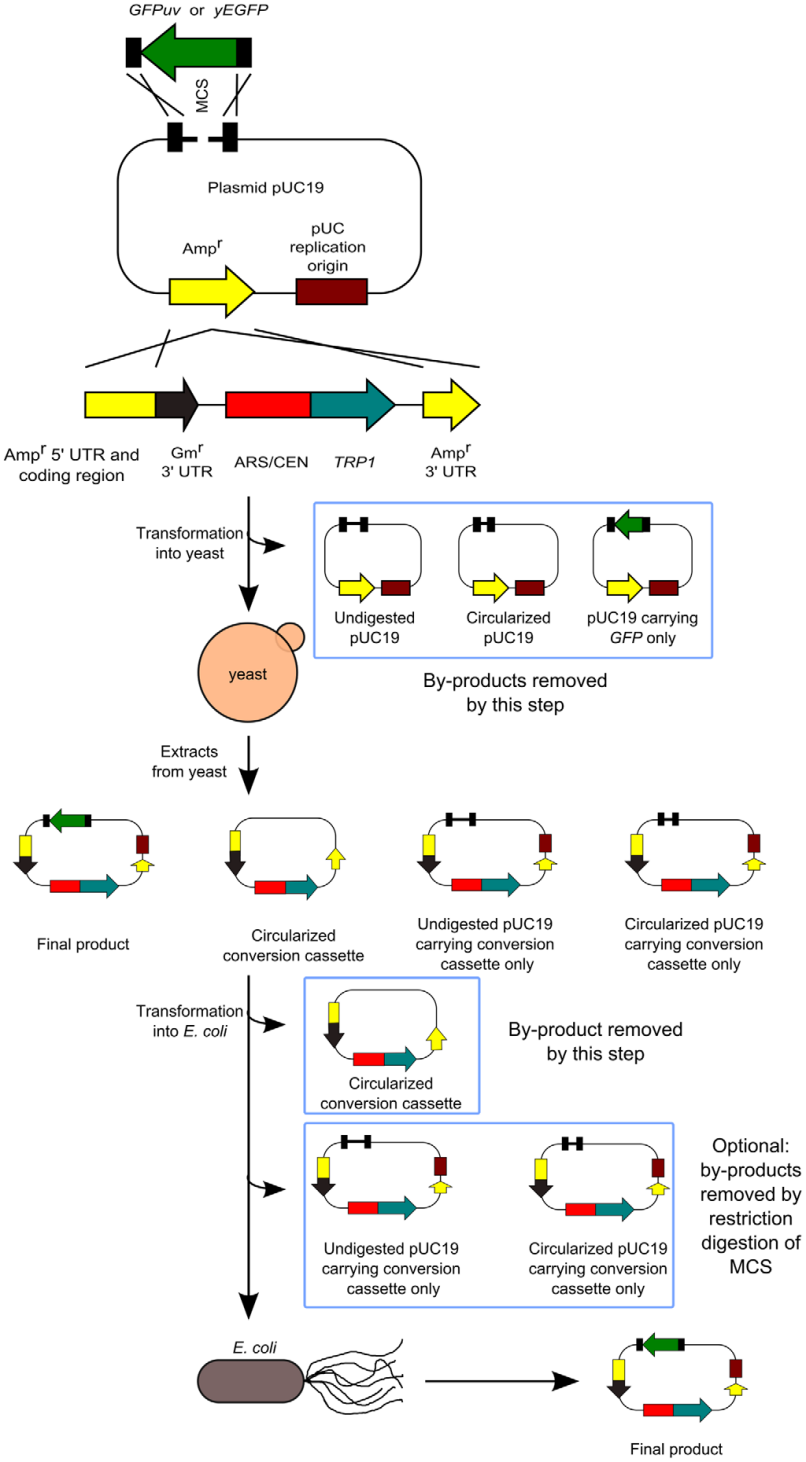
Schematic representation of the conversion of *E*.* coli* plasmid vector into a yeast/*E. coli* shuttle vector with the conversion cassette SU32 and simultaneous cloning of *GFPuv* or *yEGFP* into the same vector. The cross represents homologous recombination. MCS: multiple cloning site.

**Figure 4:**
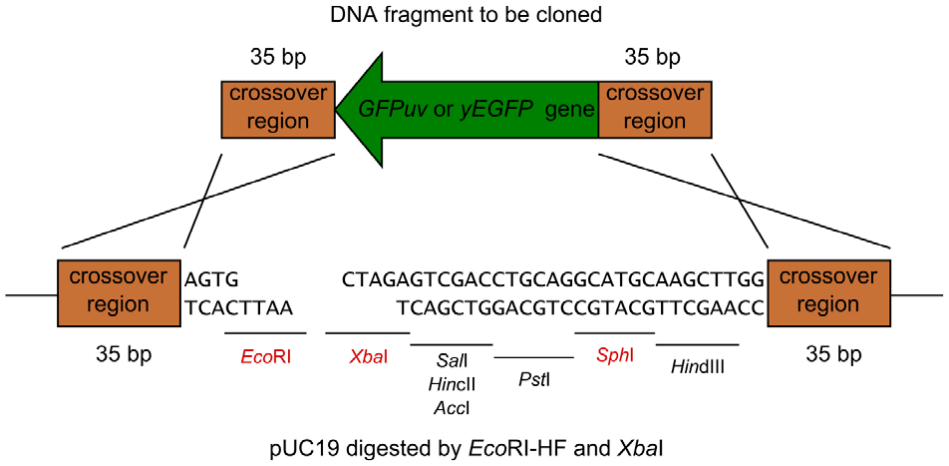
DNA sequences of the crossover regions of *Eco*RI/*Xba*I-digested pUC19 and *GFPuv*. The cross represents homologous recombination. The representative restriction sites are shown.

Next, to prove the hypothesis that we could eliminate the generation of by-products by using the new conversion cassette SU32, we cloned the *GFPuv* gene into pUC19 (Fig. 3). In this experiment, the conversion cassette SU32 converts pUC19 into the yeast/*E. coli* shuttle vector and *GFPuv* is simultaneously cloned into pUC19 by homologous recombination. pUC19 was digested with *Eco*RI-HF and *Xba*I to create suitable crossover regions. The crossover regions are not located at the restriction ends in the vector (Fig. 4); this feature is important for eliminating the generation of by-products as shown below. The green fluorescent protein (*GFPuv*) ORF was obtained by PCR using the linearized pBAD-GFPuv plasmid as a DNA template and primers that incorporate 35-bp sequences homologous to pUC19 at both ends (Fig. 4). We used PCR primers purified by polyacrylamide gel electrophoresis because a previous study [2] showed that errors in the PCR primers reduced cloning efficiency.

**Figure 5:**
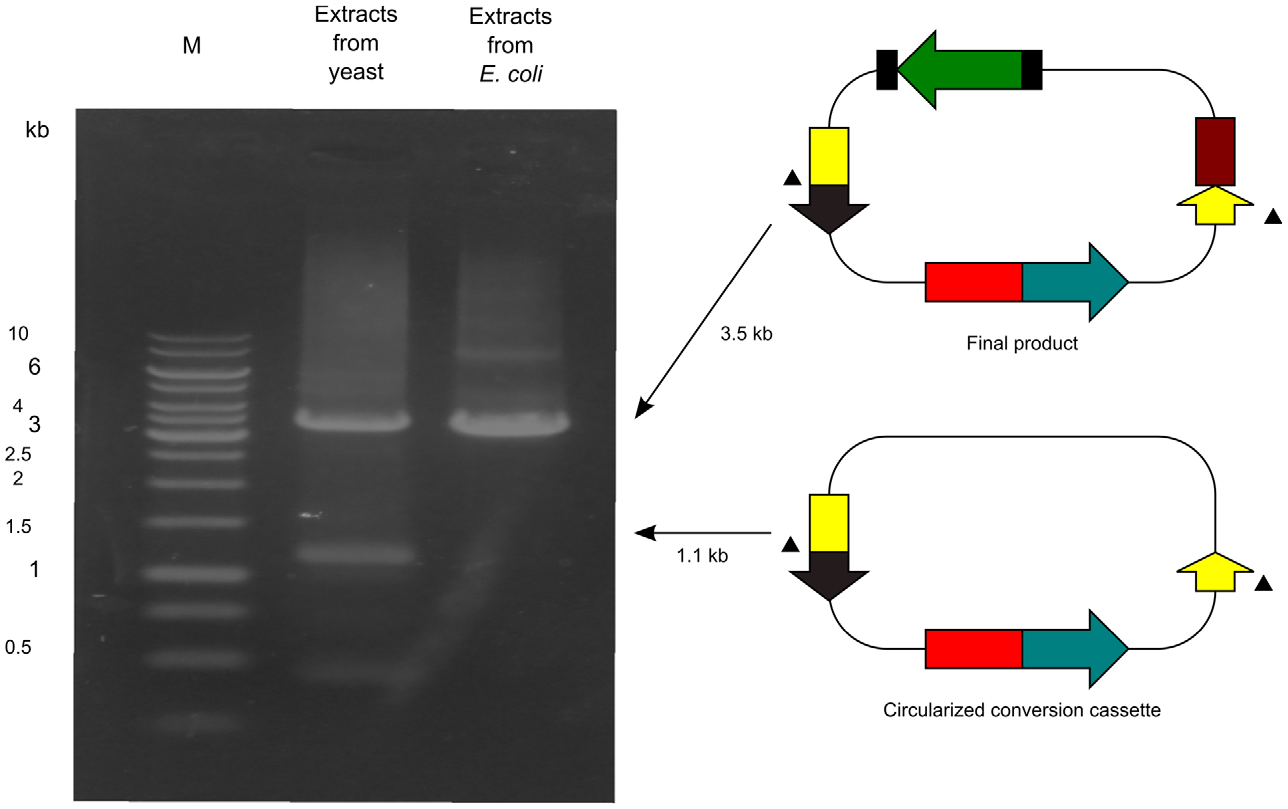
PCR analysis of the DNAs extracted from yeast. Arrowheads indicate the positions of PCR primers. GeneRuler 1-kb DNA ladder (Fermentas) is indicated on the left (M). The marker yields the following 14 fragments (in base pairs): 10,000, 8,000, 6,000, 5,000, 4,000, 3,500, 3,000, 2,500, 2,000, 1,500, 1,000, 750, 500, and 250.

The yeast culture was transformed with the cassette SU32, linearized pUC19, and *GFPuv* in a 1∶1∶1 molar ratio. This step eliminates the occurrence of the following constructs: undigested pUC19; circularized pUC19, which can be created by non-homologous end-joining in yeast; and pUC19 carrying only *GFPuv*, which can be created by homologous recombination in yeast, because these constructs carry no yeast replication origin (Fig. 3). We then rescued the plasmid DNAs from all yeast transformants. These DNAs could contain the following constructs: pUC19 carrying *GFPuv* and the conversion cassette (a final product), created by homologous recombination; a circularized conversion cassette created by non-homologous end-joining; undigested pUC19 carrying the conversion cassette only, which can be created by homologous recombination; and circularized pUC19 carrying the conversion cassette only, which can be created by homologous recombination and by non-homologous end-joining (Fig. 3). To confirm the presence of these products, PCR analysis was conducted to analyze the rescued DNAs (Fig. 5). Analysis showed the presence of the final product and the circularized conversion cassette, but no undigested or circularized pUC19 carrying the conversion cassette only. These latter products are likely present in very low quantities.

The rescued DNA was transformed into *E. coli*, and green fluorescent *E. coli* colonies were counted. This step eliminated the occurrence of circularized conversion cassette SU32, because it has no *E. coli* replication origin (Fig. 3). Thus, with approximately 99% efficiency, we obtained colonies expressing the *GFPuv* gene (Table 2, Experiments 1–3).

**Table 2:** Numbers of *E. coli* transformants carrying the insert.

	Digestion by *Sph*I	Insert	Number of *E. coli* transformants containing the insert(s)/number of tested *E. coli* transformants (percentage)
Experiment 1	No	*GFPuv*	1895/1904 (99.5%)
Experiment 2	No	*GFPuv*	2829/2839 (99.6%)
Experiment 3	No	*GFPuv*	984/1004 (98.0%)
Experiment 4	Yes	*GFPuv*	2044/2047 (99.9%)
Experiment 5	Yes	*GFPuv*	2028/2032 (99.8%)
Experiment 6	Yes	*GFPuv*	2807/2813 (99.8%)
Experiment 7	Yes	*yEGFP*	1590/1590 (100%)
Experiment 8	Yes	split *GFPuv*	624/628 (99.4%)

**Figure 6:**
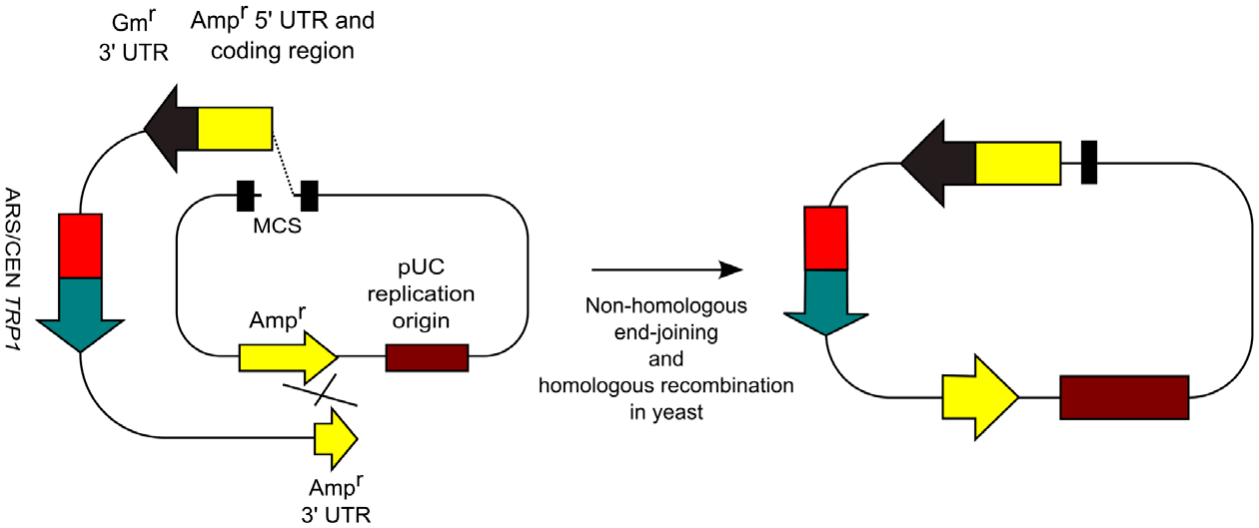
Schematic representation of the formation of a by-product. The dashed line represents non-homologous end-joining and the cross represents homologous recombination. This by-product carries part of the multiple cloning site.

To further improve the cloning efficiency, we analyzed the plasmid DNAs carried by non-*GFPuv*-expressing colonies, in other words, the by-products. One of the by-products is created by non-homologous end-joining between the restriction end of pUC19 and the 5′ end of Amp^r^ derived from the conversion cassette, and by homologous recombination of the 3′ end of Amp^r^ between pUC19 and the conversion cassette (Fig. 6). Another by-product is undigested pUC19 carrying the conversion cassette only, the presence of which has been already speculated (Fig. 3). The other by-product is pUC19 carrying a mutated *GFPuv* gene and the conversion cassette, which may be created by PCR.

**Figure 7:**
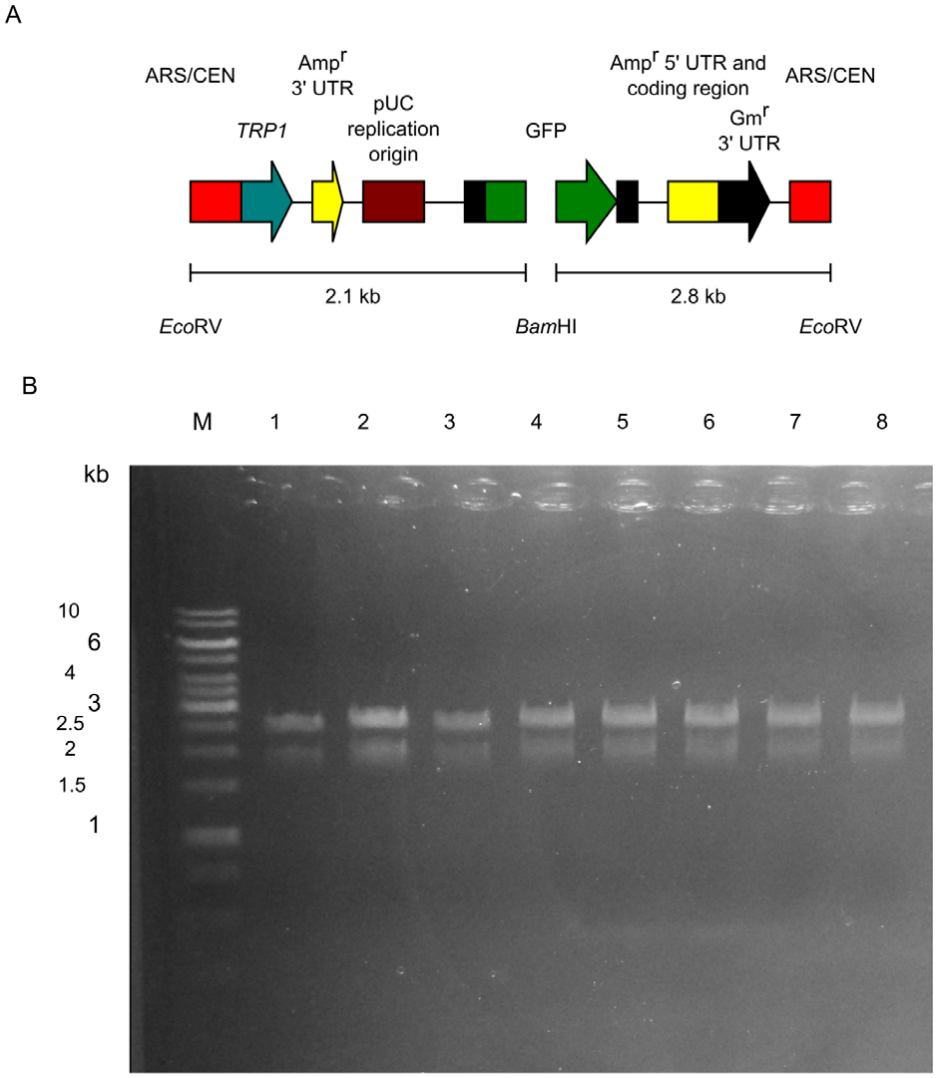
Restriction analysis of plasmids from colonies expressing *GFPuv*. Plasmids from 8 colonies expressing *GFPuv* were digested with *Eco*RV and *Bam*HI. (A) Diagram showing the restriction patterns of pUC19 carrying the conversion cassette SU32 and *GFPuv*. (B) Digested plasmids were analyzed by electrophoresis on 1% agarose gel. GeneRuler 1-kb DNA ladder (Fermentas) is indicated on the left (M).

The former 2 of 3 by-products can be eliminated by restriction digestion of DNAs isolated from yeast, because the multiple cloning site (MCS) adjacent to the restriction ends in the vector differ from the crossover regions of the vector (Fig. 3 and Fig. 4). Therefore, we digested the rescued DNAs with *Sph*I-HF and transformed them into *E. coli*. Thus, with approximately 99.8% efficiency, we obtained colonies expressing *GFPuv* (Table 2, Experiments 4–6). Restriction analysis was used to analyze 8 plasmid DNAs carried by colonies expressing *GFPuv* (Fig. 7). As expected, the *Eco*RV/*Bam*HI restriction pattern matched the predicted pattern.

**Figure 8:**
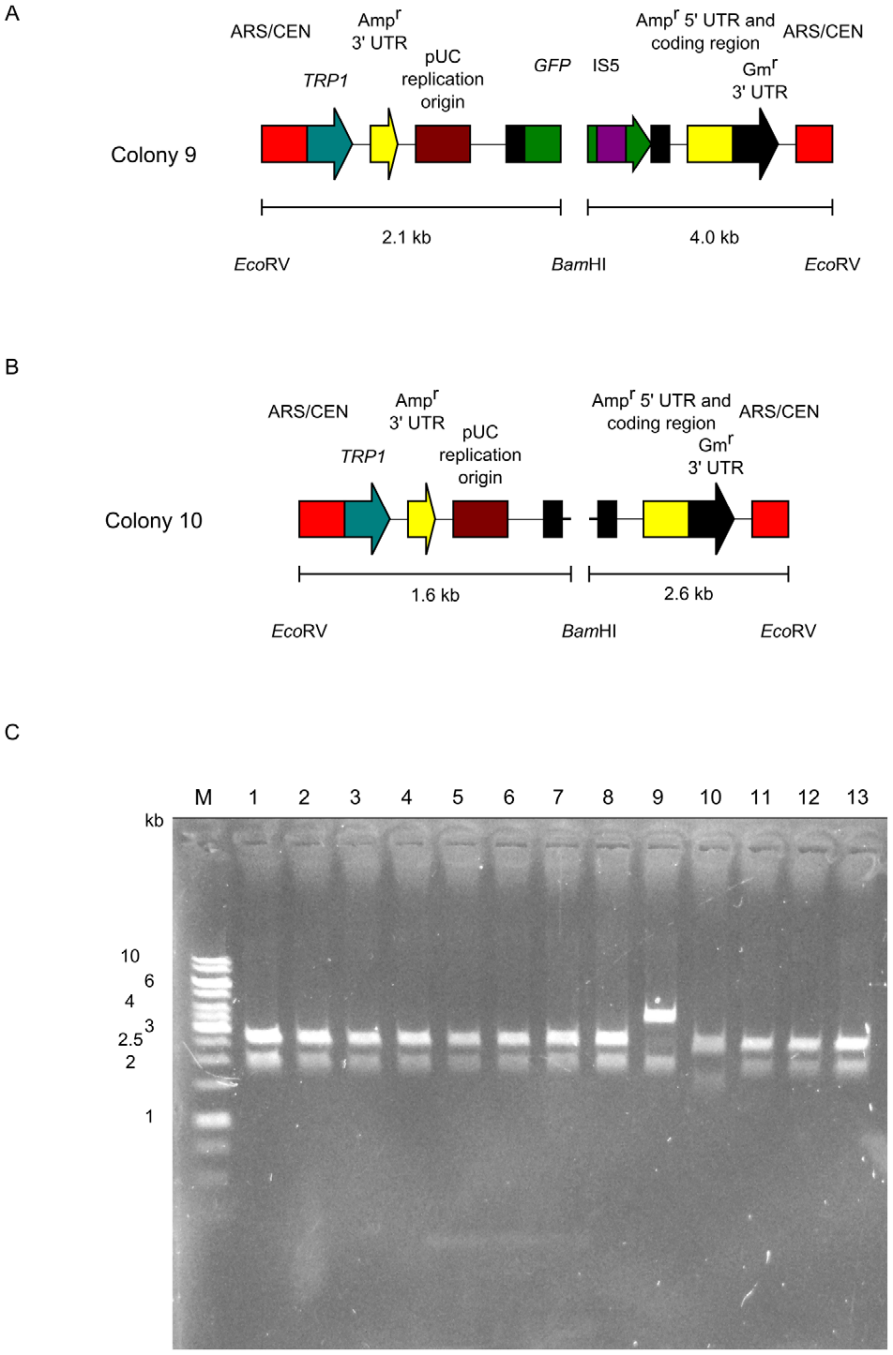
Restriction analysis of plasmids from non-*GFPuv*-expressing colonies. Plasmids from 13 non-*GFPuv*-expressing colonies were digested with *Eco*RV and *Bam*HI. (A) Diagram showing the restriction patterns of pUC19 carrying the conversion cassette SU32 and the IS5-inserted *GFPuv* gene (colony 9). IS5: transposable element in *E. coli*. (B) Diagram showing the restriction pattern of undigested pUC19 carrying the conversion cassette SU32 (colony 10). (C) Digested plasmids were analyzed by electrophoresis on 1% agarose gel. GeneRuler 1-kb DNA ladder (Fermentas) is indicated on the left (M).

Restriction analysis was used to examine 13 plasmid DNAs carried by non-*GFPuv*-expressing colonies, namely all the white colonies from Experiments 4–6. The *Eco*RV/*Bam*HI restriction pattern of 11 DNAs was the same pattern found in colonies expressing *GFPuv* (Fig. 8). DNA sequencing indicated that 10 clones carried a mutated *GFPuv* gene (Table 3). These mutations were present at sites targeted by the PCR primers. To minimize errors at these sites, we used PCR primers purified by polyacrylamide gel electrophoresis. However, the use of purified primers did not completely eliminate the emergence of errors at these sites. DNA sequencing indicated that 1 plasmid carried no mutations (Table 3, colony 8). We transformed this plasmid into *E. coli*, and the resultant colonies expressed *GFPuv*. This result indicated that a mutation in the host chromosome suppressed expression of *GFPuv*. The *Eco*RV/*Bam*HI restriction pattern of the other 2 DNAs differed from those of colonies expressing *GFPuv*. DNA sequencing and the *Eco*RV/*Bam*HI restriction pattern indicated that 1 colony had undigested pUC19 carrying the conversion cassette only (Fig. 8B) (Table 3, colony 10). This DNA likely escaped from the restriction digestion by *Eco*RI-HF, *Xba*I, and *Sph*I-HF. DNA sequencing showed that another clone carried the IS5-inserted *GFPuv* gene (Fig. 8A) (Table 3, colony 9). IS5 is one of the transposable elements in *E. coli*. The target site for IS5 in this plasmid is 5′-CTAG, consistent with previous reports [11], [12]. PCR analysis showed that the *E. coli* strain used in this study carried IS5 (data not shown). Because our cloning method is extremely efficient, we encountered these 2 rare events (colony 8 and 9). The above results showed that the primary reason for the emergence of non-*GFPuv*-expressing colonies is mutation at sites targeted by the PCR primers.

**Table 3:** Analysis of non-*GFPuv*-expressing colonies.

Colony number	Experiments	Reasons for not expressing *GFPuv*
1	Experiment 4	Error at the sites targeted by the PCR primer (717–719 ATA in *GFPuv* was changed to TTC)
2	Experiment 4	Error at the sites targeted by the PCR primer (G was inserted after 16G in *GFPuv*)
3	Experiment 4	Error at the sites targeted by the PCR primer (4G in *GFPuv* was deleted)
4	Experiment 5	Error at the sites targeted by the PCR primer (C was inserted after 5C in *GFPuv*)
5	Experiment 5	Error at the sites targeted by the PCR primer (G was inserted after 16G in *GFPuv*)
6	Experiment 5	Error at the sites targeted by the PCR primer (7A in *GFPuv* was deleted)
7	Experiment 5	Error at the sites targeted by the PCR primer (G was inserted after 4G in *GFPuv*)
8	Experiment 6	Uncharacterized mutation(s) in *E. coli* chromosome
9	Experiment 6	IS5 was inserted after 538G in *GFPuv* and before 535C in *GFPuv*
10	Experiment 6	Undigested pUC19 carrying conversion cassette only (Escape from the restriction digestions)
11	Experiment 6	Error at the sites targeted by the PCR primer (709C in *GFPuv* was deleted)
12	Experiment 6	Error at the sites targeted by the PCR primer (7A in *GFPuv* was deleted)
13	Experiment 6	Error at the sites targeted by the PCR primer (709C in *GFPuv* was deleted)

**Figure 9:**
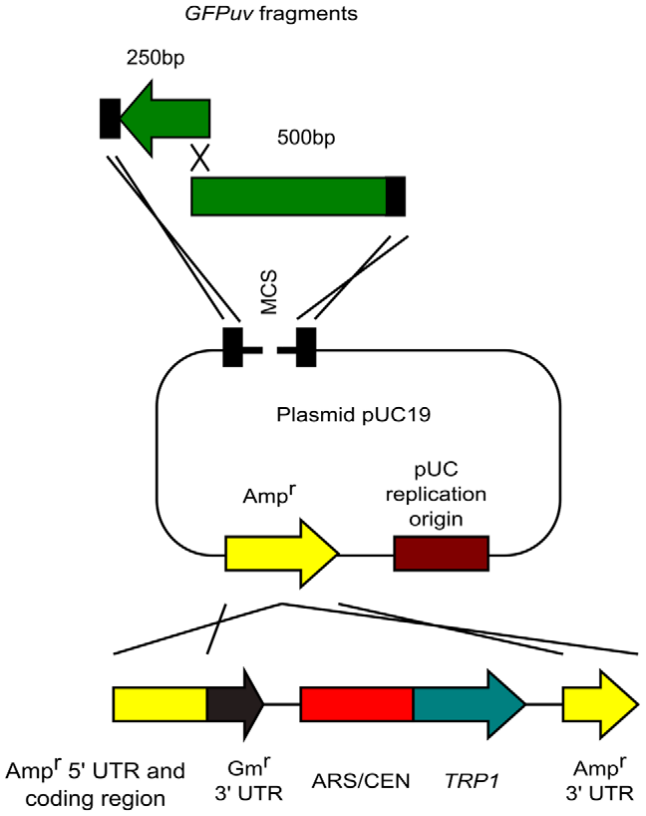
Schematic representation of the conversion of the *E.* *coli* plasmid vector into a yeast/*E. coli* shuttle vector with the conversion cassette SU32 and simultaneous cloning of 2 fragments of split *GFPuv* gene into the same vector (Experiment 8). The cross represents homologous recombination. MCS: multiple cloning site.

To further evaluate our method, we conducted another cloning experiment with the *yEGFP* gene [13], [14] and the pUC19 vector and obtained 100% cloning efficiency (Table 2, Experiment 7). This excellent result is likely due to improved primer quality. Next, we performed a multifragment cloning experiment using 2 fragments of the split *GFPuv* gene and the pUC19 vector (Fig. 9) and obtained 99.4% cloning efficiency (Table 2, Experiment 8). Thus, our method can be used for multifragment cloning.

Two methods can be used to evaluate cloning efficiency. One method is PCR or restriction analysis of the plasmid structure, and another is the counting of clones expressing reporter genes. One of our previous studies used the former method [3], whereas another study used the latter [2]. Our current study and previous studies [2], [3] showed that the cloning efficiency estimated by the former method tends to be higher [2], [3], because the efficiency of the latter method reflects errors in PCR, such as errors in the PCR primers. In this study, therefore, we evaluated the cloning efficiency based on more stringent criteria than those used in previously reported efficient cloning methods [15].

GeneArt High-Order Genetic Assembly Systems (Life Technologies), which is based on the advantages of yeast-based *in vivo* cloning, has been commercialized. This system can be used for several downstream applications: rapid construction of large DNA molecules, creation of genetic pathways, assembly of difficult-to-clone DNA fragments, manipulation of medically or industrially important microbial genomes from difficult-to-culture organisms, construction of modular expression vectors with interchangeable portions, and creation of knockout constructs. The GeneArt High-Order Vector Conversion Cassette (Life Technologies) is a commercially available system for the construction of the yeast/*E. coli* shuttle vector. This conversion system uses the traditional cloning method involving restriction enzyme cleavage followed by DNA joining with DNA ligase and does not use yeast-based *in vivo* cloning for this purpose. In contrast, by using our current system, we can simultaneously construct the yeast/*E. coli* shuttle vector and clone the desired DNA fragments into this vector.

We developed our method for the purpose of cloning DNA fragments into *E. coli* vectors. Many other methods have been developed for this purpose: Gateway cloning (Life Technologies), In-Fusion cloning (TAKARA BIO), the CloneEZ PCR cloning kit (GeneScript), circular polymerase extension cloning (CPEC) [16], [17], polymerase incomplete primer extension (PIPE) [18], quick and clean cloning [19], FastCloning [20], Inverse Fusion PCR cloning [21], and the versatile zero background T-vector system [15]. Among these methods, multifragment DNA cloning can be used for Gateway cloning, In-Fusion cloning, and CPEC. Some of these methods are highly efficient. For example, the background can be eliminated in CPEC by adding a stop codon in front of the coding sequence of the reporter gene in the backbone vector. Compared to these systems, our system has a disadvantage in that it requires 2 steps for transformation. However, our system also has advantages in that it does not require manipulation for assembling or joining DNA fragments in a test tube, the efficiency of which may sometimes depend on the reaction conditions or the skills of the person performing the procedure.

As discussed above, we evaluated the cloning efficiency of our method by using more stringent criteria than those used in previously reported efficient cloning methods. Thus, we have developed one of the most effective cloning methods for cloning a PCR product into a plasmid vector. Because the method has very high efficiency, several applications based on this procedure will possibly be available in the future. For example, our method will effectively enable expression-based selection of mutated recombinant proteins, as required, from the protein-expressing colonies from randomly mutated PCR constructs because the by-products of cloning reduce the efficiency of selection.

## Conclusions

Because it is difficult to completely eliminate errors in PCR, such as errors in the primers, we have developed a new method for cloning a PCR product into a plasmid vector, which we feel is one of the most effective methods currently available for this purpose. In other words, we have developed an “ultra-low background DNA cloning system.”
